# Daily use of extracorporeal CO_2_ removal in a critical care unit: indications and results

**DOI:** 10.1186/s40560-018-0304-x

**Published:** 2018-06-28

**Authors:** Hadrien Winiszewski, François Aptel, François Belon, Nicolas Belin, Claire Chaignat, Cyrille Patry, Cecilia Clermont, Elise David, Jean-Christophe Navellou, Guylaine Labro, Gaël Piton, Gilles Capellier

**Affiliations:** 1 Medical Intensive Care Unit, Besançon, France; 2 Service de Réanimation Médicale, CHU de Besançon, 25030 Besançon, France; 3 Department of Epidemiology and Preventive Medicine, School of Public Health and Preventive Medicine, Faculty of Medicine, Nursing and Health Sciences, Clayton, Australia; 4 Research Unit EA 3920 and SFR FED 4234, University of Franche Comté, Besançon, France

**Keywords:** Extracorporeal CO2 removal, Acute respiratory distress syndrome, Chronic obstructive pulmonary disease exacerbation

## Abstract

**Background:**

While outcome improvement with extracorporeal CO_2_ removal (ECCO_2_R) is not demonstrated, a strong pathophysiological rational supports its use in the setting of acute respiratory distress syndrome (ARDS) and COPD exacerbation. We aimed to describe our single-center experience of ECCO_2_R indications and outcome.

**Methods:**

Patients treated with ECCO_2_R in our medial ICU, from March 2014 to November 2017, were retrospectively enrolled. Primary end point was evolution of ventilator settings during the two first days following ECCO_2_R start.

**Results:**

Thirty-three patients received ECCO_2_R. Seventeen were managed with Hemolung®, 10 with Prismalung®, 4 with ILA®, and 2 with Cardiohelp®. Indications for ECCO_2_R were mild or moderate ARDS (*n* = 16), COPD exacerbation (*n* = 11), or uncontrolled hypercapnia due to other causes (*n* = 6). Four patients were not intubated at the time of ECCO_2_R start. Median duration of ECCO_2_R treatment was 7 days [5–10]. In ARDS patients, between baseline and day 2, median tidal volume and driving pressure decreased from 5.3 [4.4–5.9] mL/kg and 10 [8–15] to 3.8 [3.3–4.1] mL/kg and 9 [8–11], respectively. Prone positioning was performed in 10 of the 16 patients, without serious adverse event. In COPD patients, between baseline and day 2, median ventilation minute and PaCO2 decreased significantly from respectively 7.6 [6.6–8.7] L/min and 9.4 [8.4–10.1] kPa to 5.8 [4.9–6.2] L/min and 6 [5.3–6.8] kPa. Four out of 11 COPD patients were extubated while on ECCO_2_R. Device thrombosis occurred in 5 patients (15%). Hemolysis was documented in 16 patients (48%). One patient died of intracranial hemorrhage, while on ECCO_2_R. Twenty-four patients were discharged from ICU alive. Twenty-eight day mortality was 31% in ARDS, 9% in COPD patients, and 50% in other causes of refractory hypercapnic respiratory failure.

**Conclusion:**

ECCO_2_R was useful to apply ultra-protective ventilation among ARDS patients and improved PaCO_2_, pH, and minute ventilation in COPD patients.

**Electronic supplementary material:**

The online version of this article (10.1186/s40560-018-0304-x) contains supplementary material, which is available to authorized users.

## Background

There is not yet enough data to make strong recommendation about extracorporeal CO_2_ removal (ECCO_2_R) devices, as the benefits-risks ratio is not established. Because of its low flow, this technology is unable to provide adequate extracorporeal oxygenation. However, 350 to 500 mL/min is sufficient to remove half of CO_2_ production, making ECCO_2_R an interesting tool in several situations.

First, in the setting of acute respiratory distress syndrome (ADRS), it is well established that low tidal volume and limited plateau pressure are associated with better survival [1]. Recent guidelines recommend to aim for tidal volume of 4–8 mL/kg of predicted body weight (PBW) and plateau pressure less than 30 cmH_2_O [2]. However, ventilator-induced lung injury (VILI) due to hyperinflation has been documented even with low tidal volume [3]. Because some data suggest that decreasing plateau pressure, even if it is < 30 cmH_2_O, might be associated with reduced mortality [4], using tidal volume lower than 6 mL/kg has been proposed [5]. Three studies have showed the feasibility and safety of ultra-protective ventilation, with 4 mL/kg tidal volume and plateau pressure < 25 cmH_2_O [6–8]. However, at this time, no prospective trial has demonstrated an impact on outcome.

Second, in the setting of chronic obstructive pulmonary disease (COPD) exacerbation, noninvasive ventilation is the first option [9]. Indeed, the need for invasive mechanical ventilation is associated with higher mortality [10]. By providing extracorporeal CO_2_ clearance, ECCO_2_R might decrease respiratory rate and limit auto-PEEP, resulting in reduced respiratory work. Three case-control studies have suggested that ECCO_2_R decrease the intubation rate of severe COPD exacerbation [11–13]. It might also allow an earlier extubation and rehabilitation.

Finally, ECCO_2_R might also be useful in the setting of refractory respiratory acidosis with pH < 7.20 despite usual care. For example, successful treatment of near-fatal asthma using ECCO_2_R has been reported [14].

In this monocentric retrospective cohort study, we aimed to describe indications, ventilatory settings, gas exchanges, and outcome of patients receiving ECCO_2_R in our ICU.

## Methods

### Patients

We performed a retrospective chart review of all patients admitted to our tertiary regional intensive care unit (ICU) and started on ECCO_2_R from March 2014 to November 2017. Cases were identified through a prospectively maintained electronic database.

### Ethical issues

ECCO_2_R therapy was started while all patients were on high-intensity treatment. Families were informed of the rescue treatment and the benefits-risks ratio. As a rule, all COPD patients required either to be intubated or to fail noninvasive ventilation (NIV) or refuse intubation to be started on ECCO_2_R. Four ARDS patients were involved in ongoing studies related to ECCO_2_R.

### ECCO_2_R system

Four veno-venous ECCO_2_R systems were used, including ILA® (Novalung, Germany), Hemolung® (ALung Technologies, Inc., Pittsburgh, PA, USA), Prismalung® (Baxter Healthcare/Gambro Lund, Sweden), and CardioHelp® (Maquet, Germany). CardioHelp® was the only system using two venous cannulas. Others were associated to dual-lumen catheters, usually inserted by jugular access. There was no protocol guiding the choice of ECCO_2_R device, which was let to the clinician in charge. When renal replacement therapy was required, Prismalung® was preferentially used. ECCO_2_R weaning strategy was let to clinician’s discretion.

### Data recording

Demographic data collected included age, gender, primary admission diagnosis, cause of respiratory failure, and any known comorbidities. Physiologic data collected included vasopressor therapy and renal replacement therapy. Ventilatory data included ventilator mode, respiratory rate, tidal volume, plateau pressure when available, minute ventilation, and results of daily arterial blood gases (Additional file 1). ECCO_2_R-related data included indication of extracorporeal support, type of device, blood flow, sweep gas flow, and anticoagulation level evaluated by anti-Xa activity. Ventilation settings were gathered just before starting ECCO_2_R, at 4 hourly intervals for the first 24 h, and at day 2. Arterial blood gases were recorded once a day for 48 h.

ECCO_2_R-related or potentially linked complications included bleeding, catheter or pump thrombosis, hemolysis, thrombocytopenia, obvious local infection, and bacteremia. Bleeding at catheter insertion site was considered if associated with at least one red blood cell transfusion. Because plasma-free hemoglobin assessment was not available in our center, hemolysis was defined as anemia associated to haptoglobin less than 0,1 g/L. Thrombocytopenia was defined as a platelet count inferior to 150 G/L or a decrease of more than 50% since ECCO_2_R start.

### Statistical analysis

Qualitative variables were expressed as number percentage. Quantitative variables were expressed as median and interquartile range. Comparisons between two qualitative variables were performed using the Fischer exact test. Comparisons between two quantitative variables were performed using the Wilcoxon test. For the study of the evolution of quantitative variables over time among patients, the Wilcoxon rank sum test was performed. All analyses were performed using SAS 9.4.

## Results

### Description of the overall population

Over 4 years, 33 patients received ECCO_2_R therapy, including 16 mild or moderate ARDS patients, and 11 COPD patients with severe exacerbation. The remaining six patients had refractory hypercapnic acidosis secondary to severe acute asthma (*n* = 2), nosocomial pneumonia, bronchiolitis obliterans, exacerbation of pulmonary fibrosis, and bilateral bronchial compression by germinal tumor. Among ARDS patients who received ECCO_2_R, three were enrolled in SUPERNOVA study (NCT02282657), and one in PRISMALUNG study [15]. All COPD patients except one were intubated. Among COPD patients, 7 (63%) benefited from long-term oxygen therapy and 4 (36%) from noninvasive home ventilation. Baseline characteristics of the study population are shown in Table 1.

**Table 1:** Baseline characteristics

Variables	Patients (*n* = 33)
Age (years)	63 [59–68]
Gender (male/female)	20/13
Body mass index (kg/m^2^)	26 [23–30]
IGS2 score	49 [36–65]
SOFA score	
At ICU admission	7 [5–10]
At the time of ECCO_2_R start	10 [7–12]
Indication	
Mild or moderate ARDS	16 (48)
COPD exacerbation	11 (33)
Other	6 (19)
Associated organ dysfunction	
Maximum noradrenaline dose (μg/kg/min)	0.16 [0.00–0.25]
Need for renal replacement therapy	7 (21)
Comorbidities	
Chronic obstructive pulmonary disease	16 (48)
Arterial hypertension	22 (66)
Coronary artery disease	7 (21)
Cardiac insufficiency	6 (18)
Chronic renal impairment	2 (6)
Stroke	2 (6)
Obesity	9 (27)
Ongoing treatments	
Antiplatelet therapy	10 (30)
Anticoagulation	4 (12)
Home oxygen therapy	11 (33)
Home noninvasive ventilation	4 (12)

Numbers are *n* (%) and median [interquartile range]

### Outcome

For the 29 invasively ventilated patients, ECCO_2_R therapy was started after a median time of 1 [1–5] day of invasive mechanical ventilation. In the overall population, median duration of ECCO_2_R therapy was 7 [5–10] days, with no obvious difference between ARDS and COPD patients. Twenty-one patients were weaned from ECCO_2_R while still intubated. Median duration of invasive mechanical ventilation after ECCO_2_R weaning was 2 [0–6] days. Four COPD patients were extubated while on ECCO_2_R therapy. Only 2 ARDS patient have benefited from a tracheostomy for respiratory support weaning. Mortality at day 28 was 27% in the whole cohort and seemed to be higher in ARDS patients than in COPD patients (31 vs 9%). Three of the 6 patients with refractory respiratory acidosis died. Median length of stay in ICU was 16 [10–22] days (Table 2).

**Table 2:** Outcomes of the 33 patients receiving ECCO_2_R

Outcome	Total (*n* = 33)	ARDS (*n* = 16)	COPD (*n* = 11)	Others (*n* = 6)
28-day mortality	9 (27)	5 (31)	1 (9)	3 (50)
Length of stay in ICU, days	16 [10–22]	18 [11–26]	14 [11–19]	11 [8–18]
Duration of invasive ventilation before ECCO2R, days	1 [1–5]	3 [1–5]	1 [1–3]	0 [0–0]
Duration of ECCO2R therapy	7 [5–10]	6 [5–9]	7 [5–11]	6 [5–15]
Prone positioning, number of patients	15 (45)	10 (62)	3 (27)	2 (33)
Duration of invasive ventilation after ECCO2R weaning	2 [0–6]	4 [2–10]	2 [0–4]	1 [0–2]
Extubated while on ECCO2R, number of patients	4 (12)	0 (0)	4 (36)	0 (0)

Numbers are *n* (%) and median (interquartile range)

### Description of ECCO_2_R therapy in ARDS patients

Results of ECCO2R therapy in ARDS patients are shown in Table 3. Median baseline tidal volume, plateau pressure, driving pressure, and respiratory rate were 5.3 [4.4–5.9] mL/kg, 26 [24–27] cmH_2_O, 10 [8–15] cmH_2_O, and 26 [22–28] respectively. Twenty-four hours after ECCO_2_R start, tidal volume significantly decreased to 3.9 [3.5–4.2] mL/kg, without increase of PaCO_2_. Although there was no difference for plateau pressure, driving pressure significantly decreased to 7 [6–10] cmH_2_O. The decrease of respiratory rate was not significant. However, minute ventilation significantly decreased to 4.6 [3.9–5.8] L/min. Neuromuscular blockers were respectively used in 94, 81, and 56% of patients at baseline, day 1, and day 2. Among the 16 ARDS patients, 10 were proned while on ECCO_2_R support. Among those 10 patients, two supported by ILA®, one by Hemolung®, and one by Prismalung® had three sessions of prone positioning or more.

**Table 3:** Evolution of ventilatory settings and gas exchanges in 16 ARDS patients

Ventilatory parameters	Baseline	4 h	8 h	12 h	24 h	48 h
Ventilatory mode (VC/PSV), number	16/0	16/0	16/0	16/0	15/1	14/2
TV (mL kg^−1^)	5.3 [4.4–5.9]	3.8 [3.5–4.1]*	3.7 [3.5–4.1]*	3.7 [3.6–4.1]*	3.9 [3.5–4.2]*	3.8 [3.3–4.1]*
RR (min^−1^)	26 [22–28]	23 [20–25]	22 [19–25]*	22 [20–25]*	21 [18–23]*	23 [17–26]
Minute ventilation (L min^−1^)	8.5 [6.0–9.5]	5.2 [4.0–6.1]*	4.5 [3.9–6.3]*	4.3 [3.9–6.3]*	4.6 [3.9–5.8]*	5.3 [4.0–6.9]*
PEEP (cmH_2_O)	13 [10–15]	14 [10–15]	14 [10–18]	14 [10–18]	14 [12–18]	14 [11–17]
Plateau pressure (cmH_2_O)	26 [24–27]	–			26 [22–29]	25 [22–27]
Driving pressure (cmH_2_0)	10 [8–15]	–			7 [6–10]*	9 [8–10]*
PaCO_2_ (kPa)	6.7 [6.1–7.5]	5.7 [5.1–7.0]*			5.6 [4.8–7.6]	5.4 [4.8–8.2]
pH	7.31 [7.25–7.41]	7.39 [7.30–7.42]			7.40 [7.33–7.45]	7.41 [7.35–7.43]
PaO_2_/FiO_2_	145 [116–161]	207 [127–226]*			182 [149–211]	201 [168–263]*
Neuromuscular blockers use, number	15 (94%)	–			13 (81%)	9 (56%)

*p < 0.05 vs baseline; *V* volume control, *PSV* pressure support ventilation; numbers are *n* (%) and median (interquartile range)

Considering ultra-protective ventilation when tidal volume was ≤ 4 mL/kg of PBW and plateau pressure ≤ 25 cmH_2_O, ECCO_2_R did not allow a complete target attainment. Indeed, at baseline, 3/16 patients (19%) were ventilated with tidal volume ≤ 4 mL/kg of PBW. Plateau pressures at baseline was known for only 12/16 patients (75%) and was ≤ 25 cmH_2_O in 6/12 (50%). At baseline, only 1/12 patient (8%) has both tidal volume ≤ 4 mL/kg of PBW and plateau pressures ≤ 25 cmH_2_O (Additional file 1). Twenty-four hours after ECCO_2_R beginning, 8/16 patients (50%) were ventilated with tidal volume ≤ 4 mL/kg of PBW, 5/12 (42%) with plateau pressures ≤ 25 cmH_2_O, and 2/12 (17%) with both tidal volume ≤ 4 mL/kg of PBW and plateau pressures ≤ 25 cmH_2_O.

### Description of ECCO_2_R therapy in COPD patients

Results of ECCO_2_R therapy in COPD patients are shown in Table 4. Median baseline tidal volume, respiratory rate, minute ventilation, and PaCO_2_ were 5.5 [5.5–5.9] mL/kg; 22 [20–23]; 7.6 [6.6–8.7] L/min; and 9.4 [8.4–10.1] kPa respectively. Forty-eight hours after ECCO_2_R start, minute ventilation significantly decreased to 5.8 [4.9–6.2] L/min, while PaCO_2_ significantly decreased to 6 [5.3–6.8] kPa. Sedation was used in 72, 54, and 45% of patients at baseline, day 1, and day 2 respectively.

**Table 4:** Evolution of ventilatory settings and gas exchanges in 11 COPD patients

Ventilatory parameters	Baseline	4 h	8 h	12 h	24 h	48 h
Ventilatory mode (VC/PSV/NIV), number	8/2/1	7/3/1	7/3/1	7/3/1	5/5/1	5/5/1
TV (mL kg^−1^)	5.5 [5.5–5.9]	5 [4.4–6.1]	4.6 [4.4–4.8]*	4.5 [4.2–5]*	5.6 [4.4–5.8]	5.2 [4.1–5.8]
RR (min^−1^)	22 [20–23]	18 [17–22]	18 [15–24]	18 [15–21]	20 [17–20]	20 [16–24]
Minute ventilation (L min^−1^)	7.6 [6.6–8.7]	5.9 [5–7.2]	4.7 [3.8–5.2]*	4.9 [3.8–5.9]*	6.2 [4.1–6.8]	5.8 [4.9–6.2]*
PEEP (cmH_2_O)	6 [4–8]	6 [5–8]	6 [5–8]	6 [5–8]	8 [5–10]	8 [7–10]
PaCO_2_ (kPa)	9.4 [8.4–10.1]	6.6 [5.7–7.1]*			6 [5.1–7.1]*	6 [5.3–6.8]*
pH	7.32 [7.26–7.34]	7.43 [7.41–7.45]*			7.45 [7.41–7.47]*	7.44 [7.42–7.46]*
PaO_2_/FiO_2_	174 [158–207]	192 [177–254]			225 [186–262]	210 [181–254]
Sedative use, number	8 (72%)	– –			6 (54%)	5 (45%)
Neuromuscular blockers use, number	6 (54%)				4 (36%)	3 (27%)

*p < 0.05 vs baseline; *VC* volume control *PSV* pressure support ventilation, *NIV* noninvasive ventilation; ^1^when VC is used; numbers are *n* (%) and median (interquartile range)

### Description of ECCO_2_R therapy in non-intubated patients

Only four patients were not intubated at the beginning of ECCO_2_R therapy. Patient no. 1 was a 61-year-old man who had a history of kidney transplantation. He had refractory hypercapnic COPD exacerbation with NIV failure. He received 2 days of ECCO_2_R by Hemolung® device until successful weaning without need for intubation. Patient no. 2 was a 90-year-old woman with refractory hypercapnic pneumonia without hypoxemia. She was weaned from Prismalung® device after 5 days, but she died 48 h later in a context of withdrawal of active treatments. Patient no. 3 was a 59-year-old man with a history of lung transplantation and chronic graft rejection. He was assisted by Hemolung®. As he was not eligible for re-transplantation, he died at day 19 while still on ECCO_2_R, in a context of withdrawal of active treatments. Patient no. 4 was a 29-year-old man with microscopic polyangeitis with end-stage renal failure associated to lung fibrosis. He benefited from Prismalung® at day 0. He was intubated at day 6 and died at day 17 of septic shock.

### ECCO_2_R devices

Among the 33 enrolled patients, 17 were treated with Hemolung®, 10 with Prismalung®, 4 with ILA, and 2 with Cardiohelp®. Hemolung® was used in 9 of 11 COPD patients, whereas Prismalung® was used in 6 of 16 ARDS patients. ILA® and Cardiohelp® were used only in ARDS patients. Evolution of anti-Xa activity and platelet levels are reported in Table 5.

**Table 5:** Hemostatic parameters during ECCO_2_R therapy

Devices	Duration of therapy (days)	Platelets count (G/L)						Anti-Xa activity (UI/mL)						Hemolysis	RBC transfusion during ECCO_2_R
		J0	J1	J2	J3	J4	J5	J0	J1	J2	J3	J4	J5		
All patients (*n* = 33)	7 [5–10]	202 [137–286]	167 [95–243]	143 [88–199]	130 [79–192]	128 [89–156]	124 [86–153]	0.22 [0.14–0.47]	0.26 [0.16–0.40]	0.33 [0.24–0.44]	0.33 [0.25–0.41]	0.30 [0.20–0.39]	0.35 [0.21–0.47]	16 (48)	16 (48)
Hemolung® (*n* = 17)	7 [5–10]	210 [163–264]	184 [108–243]	136 [88–208]	121 [79–174]	128 [89–153]	130 [88–151]	0.24 [0.15–0.50]	0.26 [0.20–0.36]	0.41 [0.30–0.53]	0.31 [0.25–0.42]	0.30 [0.20–0.38]	0.35 [0.30–0.49]	13 (76)	9 (53)
Prismalung® (*n* = 10)	5 [3–6]	153 [104–272]	126 [65–241]	124 [72–195]	105 [57–183]	82 [57–98]	77 [55–87]	0.13 [0.10–0.43]	0.17 [0.10–0.24]	0.25 [0.10–0.41]	0.37 [0.14–0.40]	0.23 [0.12–0.35]	0.20 [0.11–0.36]	1 (10)	4 (40)
ILA® (*n* = 4)	10 (5–19)*	226 [150–288]	181 [155–225]	172 [142–206]	186 [167–208]	151 [134–184]	173 [146–199]	0.19 [0.16–0.28]	0.36 [0.29–0.39]	0.26 [0.25–0.29]	0.37 [0.32–0.42]	0.31 [0.24–0.34]	0.26 [0.22–0.31]	1 (25)	3 (75)
Maquet® (*n* = 2)	6.5 (6–7)*	258 [205–311]	179 [163–195]	151 [141–162]	142 [130–153]	142 [130–153]	153 [153–153]	0.27 [0.25–0.30]	0.49 [0.44–0.53]	0.47 [0.46–0.47]	0.45 [0.42–0.47]	0.58 [0.55–0.61]	0.47 [0.46–0.47]	1 (50)	0 (0)

Numbers are *n* (%) and median (interquartile range), except for * which corresponds to min and max. *RBC* red blood cells

### Complications

No decannulation was reported during the studied period. Thrombocytopenia was the most frequently reported adverse event (72%). Since day 2 of ECCO_2_R treatment, at least half of treated patients have less than 150 G/L of platelets and at least 25% have less than 90 G/L of platelets. However, only four patients (12%) received platelet transfusion. Half of the treated patients received at least one red blood cell transfusion during ECCO_2_R therapy. One patient treated with Hemolung® died of intracranial hemorrhage, while on ECCO_2_R. At the diagnosis, he had 189 G/L of platelets, and 0,44 UI/mL of antiXa. Hemolysis was reported in 16 patients (48%) but did not lead to ECCO_2_R withdrawal. Device thrombosis occurred in 5 patients (15%). Among them, one ARDS patient treated with CardioHelp® has necessitated urgent circuit change for complete pump thrombosis. Interruption of CO_2_ removal for ECCO_2_R change or withdrawal was well tolerated in all cases.

## Discussion

In this retrospective chart review, we aimed to describe our experience of ECCO_2_R devices and to help clinician volunteers to use those devices beyond the scope of experimental studies. We have found that ECCO_2_R system allowed ultra-protective ventilation in ARDS patients by decreasing tidal volume. We also found that ECCO_2_R was effective to reduce minute ventilation and improve blood pH in ventilated COPD patient. Furthermore, it allowed extubation in some patients while on extracorporeal support.

In the setting of ARDS patients, our results are in line with those of the three main studies assessing veno-venous low-flow ECCO_2_R. Indeed, in a recent pilot study in 15 mild to moderate ARDS patients, the use of Hemolung® allowed ultra-protective ventilation with tidal volume of 4 mL/kg [8]. Some similar results have been observed with Polystan SAFE® (Maquet, Rastatt, Germany), a membrane lung connected to a veno-venous hemofiltration system [6]. Interestingly, in our ARDS cohort, 6 of the 16 patients have benefited from Prismalung® device, connected to a renal replacement machine. In a recently published proof-of-concept study, Prismalung® allowed ultra-protective ventilation in 20 mild-to-moderate ARDS patients. However, mean duration treatment was only 31 h (± 22), limiting conclusion about longer use and safety [15]. Although significant, the reduction of driving pressure secondary to tidal volume decrease was quite small in our study. This could be due to a relatively low tidal volume at baseline. Indeed, in our practice, we usually target low plateau pressure, with tidal volume lower than 6 mL/kg as reported by other authors [16]. After ECCO_2_R start, we have noted a trend to decrease of respiratory rate. This is another potential aspect of the ultra-protective strategy, as some authors have suggested that respiratory rate could be a determinant of VILI [17]. Because median baseline PaO_2_/FiO_2_ ratio was 145 [116–161] mmHg, patients were a priori susceptible to have an indication for prone positioning. It is noteworthy that 10 have benefited from this therapy without any adverse event. Because prone positioning has been found to decrease mortality in ADRS with PaO_2_/FiO_2_ ratio less than 150 mmHg, it is mandatory that novel therapy do not limit its use [18]. While neuromuscular blockers use in moderate to severe ARDS is recommended for 48 h [19], we have noted a trend towards early interruption. This might be explained by the early improvement of oxygenation and the control of hypercapnia. In this situation, our clinicians favored decrease sedations and awakeness.

In the three largest studies enrolling COPD patients, the primary end point was the avoidance of intubation [11–13]. In our cohort, only one COPD patient has benefited from such pre-intubation strategy. However, whether ECCO_2_R should be started before or after intubation is still a matter of debate. Indeed, because NIV failure is hard to predict, and ECCO_2_R is associated with additional septic and hemorrhagic risks, appropriate selection of patients which might benefit from ECCO_2_R is difficult. Considering this, our policy is to start ECCO_2_R early after intubation. Moreover, such strategy may facilitate insertion of the ECCO_2_R catheter, allowing safe conditions, as no complication was reported during catheter insertion in our patients. In our cohort, 50% of the patients were started on day 1 and 75% before day 3 of mechanical ventilation. While the global effect of ECCO_2_R was the lowering of minute ventilation and PaCO_2_, in our cohort, it was more marked during the 24 first hours. This might be potentially explained by the decrease of sedative use, resulting in more patients with spontaneous ventilation, with uncontrolled tidal volume and respiratory rate.

Considering safety, our data point out that ECCO_2_R use cannot be dissociated from the potential risks of anticoagulation in critically ill patients. Indeed, as it occurred during ECCO_2_R therapy, the fatal case of intracranial hemorrhage has to be underlined. Even if no heparin overdosing has been reported, there is an obvious link between anticoagulation and this complication. Even if the hemorrhagic risk was not obvious, as there was no other indication for anticoagulation, responsibility of ECCO_2_R therapy is here plausible. Otherwise, although frequent, thrombocytopenia was moderate, and hemolysis did not result in need for ECCO_2_R withdrawal. Finally, because interruption of CO_2_ removal was well tolerated, ECCO_2_R circuit thrombosis only resulted in loss of 250 mL of blood, corresponding to the circuit volume of purge.

Because, no trial demonstrating its clinical benefit has been published, ECCO_2_R systems are not widely used. Indeed, in a recent survey, among 239 French ICUs, only 15% declared having used at least once ECCO_2_R between 2010 and 2015 [20]. However, ECCO_2_R technology has improved, and because of a strong rational, several randomized trials enrolling ARDS and COPD patients are ongoing [21]. The mortality rate in our ARDS and COPD patients is in the lower range [16, 22], and early use of ECCO2R might contribute to our results. If well-designed studies bring proof of the ECCO_2_R benefit, a very large number of patients would be concerned, asking the question of where to perform ECCO_2_R. Indeed, in the setting of ECMO, a large retrospective cohort analysis has suggested a negative link between ECMO cases volume and hospital mortality [23]. As well as the concept of “ECMO center,” the need for “ECCO_2_R center” has to be assessed.

Our study has several limitations. First, because of the retrospective design, some data are lacking. Whereas tidal volume, respiratory rate, and positive end expiratory pressure are monitored hourly by the nurses in our unit, plateau pressure is usually monitored by the clinicians and not systematically reported in the medical record. It explains why complete data on plateau pressure and driving pressure were available for only 8 on 16 ARDS patients. Second, heterogeneity and small sample size limit internal validity. However, ECCO_2_R is not widely used, and previous studies in the setting of ECCO_2_R have included no more than 40 ARDS patients [7] and 25 COPD patients [12, 13]. Third, we reported a single-center experience limits the generalization of our conclusions. For example, our paramedical team is widely used to prone positioning and extracorporeal circulations. Even if prone positioning in patients receiving ECCO_2_R has only concerned a few patients, team practice might have decreased the risk of severe adverse events such as accidental decannulation. Our patients represent 256 ECCO_2_R days. Fourth, except in the setting of SUPERNOVA or PRISMALUNG studies, we did not have preset criteria for ECCO_2_R implantation, which was left to the clinician’s judgment. Fifth, we reported only initial ECCO_2_R settings. However, because of maximal CO_2_ is targeted, sweep gas was usually kept at his maximal value. ECCO_2_R rotation per minute (RPM) was set to reach a blood flow of at least 300, 450, 700 and 1000 mL/min, with Prismalung®, Hemolung®, ILA®, and CardioHelp® respectively. ECCO_2_R RPM were decreased only when significant hemolysis was documented. Finally, plasma-free hemoglobin assessment was not available in our center, resulting in a more difficult diagnosis of hemolysis.

## Conclusion

In ARDS patients, ECCO_2_R use was associated with a significant decrease of tidal volume and driving pressure during the first 48 h of therapy. Prone positioning was performed in 10 (62%) patients without adverse event. In COPD patients, ECCO_2_R use was associated with a significant decrease of minute ventilation, normalization of pH, and decrease of PaCO_2_. Although ECCO_2_R therapy was globally well tolerated, a case of fatal intracranial hemorrhage points out that this procedure cannot be dissociated from the potential risks of anticoagulation in critically ill patients.

## Additional file

Additional file 1: **Figure S1.** Scatter plots representing evolution of tidal volume (a), respiratory rate (b), plateau pressure (c) and driving pressure (d) of the 16 ARDS patients. (DOCX 80 kb)
