# Supplementary material for: Daily use of extracorporeal CO2 removal in a critical care unit: indications and results
Source: J Intensive Care. 2018 Jun 28;6:36. doi: 10.1186/s40560-018-0304-x (PMC6022441; doi:10.1186/s40560-018-0304-x)
Supplement: Supplementary file 1 — Figure S1. Scatter plots representing evolution of tidal volume (a), respiratory rate (b), plateau pressure (c) and driving pressure (d) of the 16 ARDS patients. (DOCX 80 kb) [file 40560_2018_304_MOESM1_ESM.docx]

Supplementary material 1 : Scatter plots representing evolution of tidal volume (a), respiratory rate (b), plateau pressure (c) and driving pressure (d) of the 16 ARDS patients


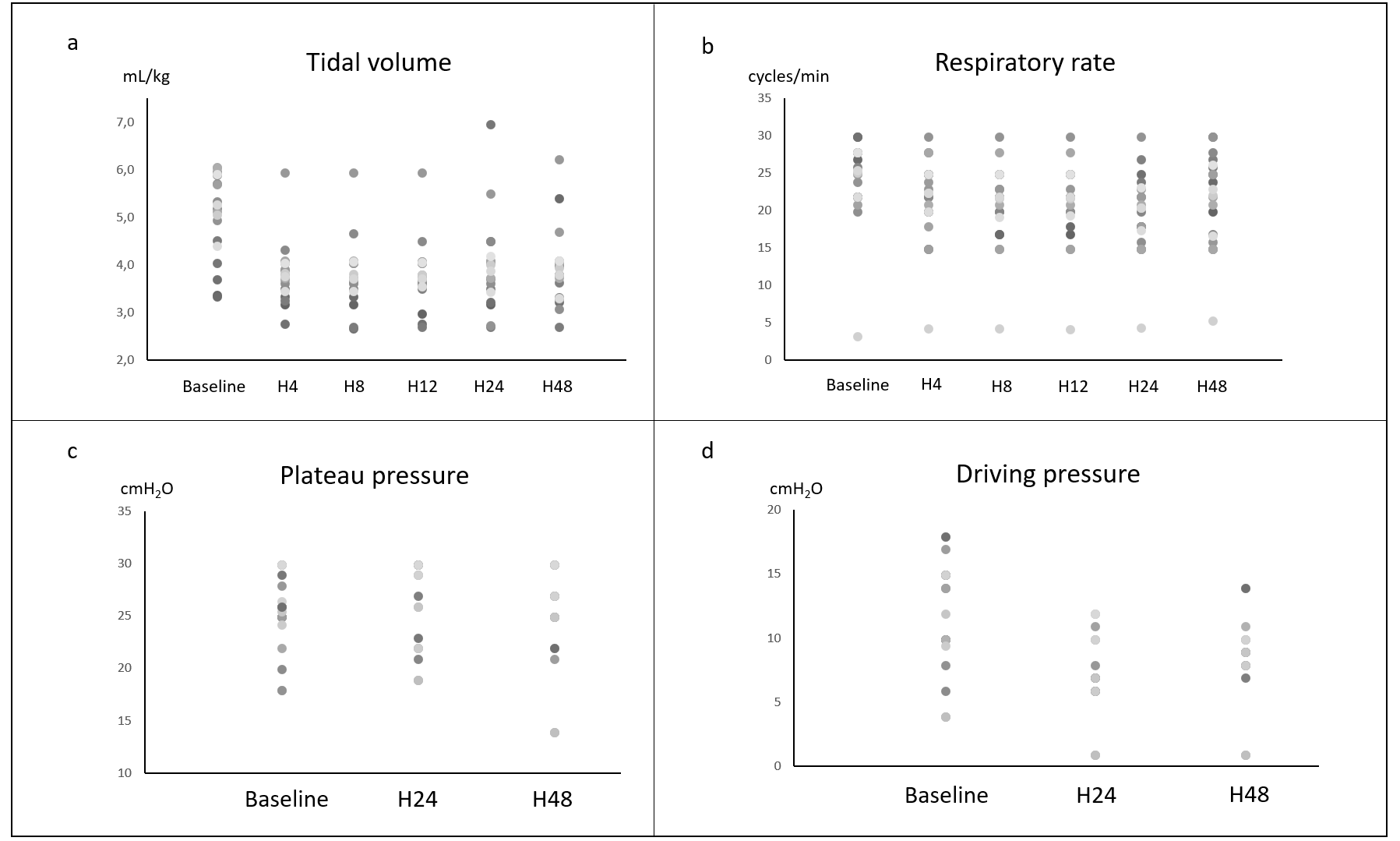


Plateau pressure and driving pressure values were only available for 12 patients
